# γ-Core Guided Antibiotic Design Based on Human Enteric Defensin 5

**DOI:** 10.3390/membranes13010051

**Published:** 2022-12-31

**Authors:** Gaomei Zhao, Changsheng Jia, Cheng Zhu, Minchao Fang, Chenwenya Li, Yin Chen, Yingjuan He, Songling Han, Yongwu He, Jining Gao, Tao Wang, Cheng Wang, Junping Wang

**Affiliations:** 1 State Key Laboratory of Trauma, Burns and Combined Injury, Institute of Combined Injury of PLA, Chongqing Engineering Research Center for Nanomedicine, College of Preventive Medicine, Third Military Medical University, Chongqing 400038, China; 2 Department of Pharmacy, Southwest Hospital, Third Military Medical University, Chongqing 400038, China; 3 Tianjin Key Laboratory of Function and Application of Biological Macromolecular Structures, School of Life Sciences, Tianjin University, Tianjin 300072, China

**Keywords:** human defensin 5, γ-core structure, antibacterial action, membrane attraction, membrane destruction, MRSA, structure-activity study

## Abstract

An increase in the number of infections caused by resistant bacteria worldwide necessitates the development of alternatives to antibiotics. Human defensin (HD) 5 is an innate immune peptide with broad-spectrum antibacterial activity, but its complicated structure makes its preparation difficult. Herein, we truncated the HD5 structure by extracting the highly conserved γ-core motif. A structure-activity study showed that this motif was ineffective in killing bacteria in the absence of specific spatial conformation. Notably, after the introduction of two intramolecular disulfide bonds, its antibacterial activity was markedly improved. Glu and Ser residues were then replaced with Arg to create the derivative RC18, which exhibited stronger potency than HD5, particularly against methicillin-resistant *S. aureus* (MRSA). Mechanistically, RC18 bound to lipid A and lipoteichoic acid at higher affinities than HD5. Furthermore, RC18 was more efficient than HD5 in penetrating the bacterial membranes. Molecular dynamics simulation revealed that five Arg residues, Arg1, Arg7, Arg9, Arg15, and Arg18, mediated most of the polar interactions of RC18 with the phospholipid head groups during membrane penetration. In vivo experiments indicated that RC18 decreased MRSA colonization and dramatically improved the survival of infected mice, thus demonstrating that RC18 is a promising drug candidate to treat MRSA infections.

## 1. Introduction

In light of antibiotic misuse and the lack of progress in drug development, resistant bacteria have increasingly emerged worldwide, and they severely threaten human health [1]. As recently reported, bacterial resistance was associated with an estimated 1.27 million deaths in 2019, and the primary pathogens, including Gram-negative (G^−^) *Acinetobacter baumannii* (*A*. *baumannii*) and Gram-positive (G^+^) *Staphylococcus aureus* (*S*. *aureus*), contributed to 929,000 deaths [2]. Given the absence of new efficient therapeutics, bacterial infections caused by “superbugs” could result in approximately 10 million deaths per year by 2050, costing the global economy 100 trillion dollars annually [3]. To cope with this global crisis, antimicrobial peptides (AMPs), which are of great value in the age of resistance, have gained attention in the field of antibiotic development [4].

Defensins, a group of endogenous AMPs that are produced in response to invading microbes, including bacteria, viruses and fungi, are crucial elements of innate immunity in mammals, insects, and plants [5]. Human defensins (HDs) have a characteristic β-strand structure stabilized by three intermolecular disulfide bonds [6]. Based on sequence homology and connectivity of the Cys residues, HDs are classified into two subfamilies, α (Cys1-6/Cys2-4/Cys3-5) defensins and β (Cys1-5/Cys2-4/Cys3-6) defensins. Human α defensins were originally identified as natural effectors that are stored in neutrophil azurophilic granules [7,8]. Then, it was found that Paneth cells, which reside at the bottom of the small intestine crypt, secrete another two peptides in response to bacterial infection, i.e., HD5 and HD6 [9,10]. HD5 is a broad-spectrum antibacterial agent that is an α defensin [11]. Deficiency of the HD5-homologous peptide in the mouse intestine results in susceptibility to orally administered *Salmonella typhimurium* [12]; conversely, HD5-transgenic mice are more resistant to lethal bacterial infection [13], implying that HD5 has considerable value as an alternative to antibiotics.

HD5 is a 32-residue-long peptide with specific disulfide pairings that make its preparation difficult. Additionally, the shielding effects caused by high concentrations of saline and anionic proteins or nucleic acids in the serum attenuate the antibacterial activity of HD5 [14]. We previously conducted structure-activity studies on HD5 and discovered that sequence simplification by increasing the electropositive charge while simultaneously maintaining the evolutionarily conserved elements was instrumental to overcoming the molecule’s disadvantages [15,16]. Disulfide-stabilized natural AMPs spanning different species share a striking multidimensional signature termed the γ-core motif, which has a hallmark of GXCX_3-9_C in the sequence and consists of two antiparallel β-sheets and an interposed short turn region [17]. This conserved motif has attracted considerable attention in anti-infective drug discovery for resistant bacteria [18], pathogenic fungi [19], and even severe acute respiratory syndrome coronavirus 2 (SARS-CoV-2) [20].

In this study, we evaluated the effect of the γ-core motif on the antibacterial activity of HD5. Intramolecular disulfide bonds and Arg residues were introduced for functional improvement. A γ-core-based peptide antibiotic with potent efficacy against resistant bacteria was designed and termed RC18. Biolayer interferometry (BLI) and scanning electron microscopy (SEM) were used to investigate the underlying mechanism by which RC18 killed bacteria. A mouse intraperitoneal infection model was established to determine the antibacterial activity of RC18 in vivo. Our study demonstrates an important strategy for the design of defensin-derived antibiotics and provides a potential therapeutic alternative for resistant bacteria.

## 2. Materials and Methods

### 2.1. Peptide Synthesis

**Figure 1:**
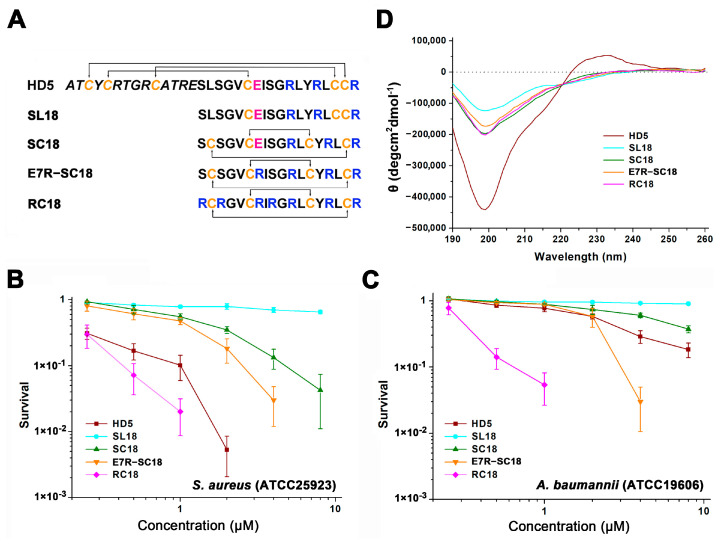
(**A**) Amino acid composition of the peptides. Cysteines are coloured orange. Glu21 is coloured pink. Arginines in the γ-core motif are coloured blue. (**B**) Virtual colony count assays were used to determine the antibacterial action of the peptides against *S. aureus* (ATCC 25923) and (**C**) *A. baumannii* (ATCC 19606). Bacterial survival was calculated as the ratio of the number of surviving colonies after treatment with peptides (0.25, 0.5, 1, 2, 4, and 8 µM) to the total number of colonies in the control group. Spots below 10^−3^ bacteria were considered totally inactive and are not shown. The results are presented as the mean ± standard deviation (SD). (**D**) CD spectra of the peptides in water.

All peptides were synthesized by Guoping Pharmaceutical Company (Hefei, Anhui, China) using solid-phase chemical synthesis. No specific modifications existed at the peptide terminus. The amino acid sequences of the peptides are displayed in Figure 1A. The peptide purities were determined with high-performance liquid chromatography (SHIMADZU LC-10AT, Kyoto, Japan). A SHIMADZU Inertsil ODS-SP column (5 μm, 4.6 × 250 mm) and a linear gradient of 15–55% buffer B (buffer A: 0.1% trifluoroacetic acid in water; buffer B: 0.1% trifluoroacetic acid in acetonitrile) were utilized for chromatography. The molecular weights were confirmed using a SHIMADZU (Kyoto, Japan) LCMS-2020 mass spectrometer (Figure S1). HD5 peptide has been characterized in our recent studies [21].

### 2.2. Virtual Colony Count Antibacterial Assay

A virtual colony count assay was used to determine the antibacterial action of the peptides in vitro as previously described [22]. Briefly, *S. aureus* (ATCC 25923) and *A. baumannii* (ATCC 19606) were grown to mid-logarithmic phase and were diluted to 1 × 10^6^ CFU/mL using sodium phosphate buffer (pH = 7.4) containing 1% Müller-Hinton broth (MHB). Peptides were prepared in sterile water at the concentrations of 2.5, 5, 10, 20, 40, and 80 µM. A total of 10 µL of peptides were incubated with 90 µL of microbes at 37 °C for 1 h. Then, another 100 µL of 2 × MHB was added, and bacterial growth was monitored at 600 nm with a M2e microplate reader (Molecular Devices, Silicon Valley, CA, USA) for 12 h. This experiment was conducted in triplicate and repeated twice.

### 2.3. Circular Dichroism (CD) Spectroscopy

The structures of HD5 and its derivatives (100 μg/mL, 250 μL) in deionized water were analyzed with an Applied Photophysics (Leatherhead, Surrey, UK) Chirascan instrument at 25 °C. A cell with a 1 mm path length was used. The spectra were detected from 190–260 nm at 1 nm intervals. The results obtained from three independent assays were processed by Pro-Data Chirascan software (version 4.1).

### 2.4. Broth Microdilution Assay

The minimum inhibitory concentrations (MICs) of RC18 and HD5 were measured using the broth microdilution method recommended by the Clinical and Laboratory Standards Institute (CLSI) with modifications made by the Hancock laboratory [23]. Peptides prepared in 0.02% acetic acid containing 0.4% bovine serum albumin (BSA) were used as the stock solution. After dilution to concentrations of 3200, 1600, 800, 400, 200, 100, and 50 μg/mL using 0.01% acetic acid containing 0.2% BSA, the peptides (11 μL) were incubated with 100 μL of 2 × 10^5^ CFU/mL bacteria, including multidrug-resistant *A. baumannii* (MDRAB) [15], *A. baumannii* (ATCC 19606), methicillin-resistant *S. aureus* (MRSA, ATCC 43300), and *S. aureus* (ATCC 25923), in a polypropylene 96-well microtiter plate. The absorbance at 600 nm was detected using a microplate reader after 24 h. Peptide concentration at which no visible bacterial growth was observed was identified as the MIC. For cefotaxime (CTX) and ciprofloxacin (CIP), the MICs were determined based on the standard broth microdilution method outlined by the CLSI.

### 2.5. Biolayer Interferometry (BLI)

The binding kinetics of RC18 to lipid A (L5399, Sigma, Shanghai, China) and lipoteichoic acid (LTA, L2515, Sigma) were measured using the ForteBio Octet Red 96 BLI platform (Sartorius BioAnalytical Instruments, Bohemia, NY, USA). The amine-reactive second-generation (AR2G) biosensors were processed with 1-ethyl-3-(3-dimethylaminopropy) carbodiimide hydrochloride (EDC, E1769, Sigma) and sulfo-N-hydroxysulfosuccinimide (s-NHS, 56485, Sigma). RC18 was diluted with 10 mM sodium phosphate buffer (pH 7.4) at concentrations ranging from 200 to 1200 nM. Lipid A and LTA were loaded onto the activated AR2G biosensors at 30°C for 5 min. Association and disassociation were both carried out for 5 min. To determine the recruitment of peptides to lipopolysaccharide (LPS), AR2G biosensors immobilized with 10 μg/mL LPS (L2880, Sigma) were incubated with 1000 nM HD5 and RC18 for 5 min. The binding thickness was recorded and analysed using ForteBio Data Analysis 7.0 software. The equilibrium dissociation constant (*K*_D_) was calculated as the dissociation rate constant (*K*_off_) divided by the association rate constant (*K*_on_)_._

### 2.6. Zeta Potential Determination

A total of 900 µL of mid-logarithmic-phase *S. aureus* (ATCC 25923) diluted to 1 × 10^8^ CFU/mL in 10 mM sodium phosphate buffer (pH = 7.4) was incubated with 100 µL of peptides or sterile water at 25 °C for 2 min and loaded into a disposable zeta cell. Bacterial surface charge was detected using a Malvern (Malvern, UK) Zetasizer Nano ZS. This assay was conducted in triplicate and repeated twice on different days.

### 2.7. NPN Uptake Assay

MDRAB was grown to mid-logarithmic phase and was diluted to 1 × 10^8^ CFU/mL in 10 mM sodium phosphate buffer (pH = 7.4). A total of 190 µL of MDRAB was exposed to 10 µL of HD5 and RC18 at 37 °C for 1 h. 1-N-phenylnaph-thylamine (NPN, 104043, Sigma, Shanghai, China) was then added at a final concentration of 10 µM. After mild shaking, the fluorescence intensity was measured from 380 to 450 nm at an interval of 2 nm following excitation at 350 nm using a Tecan Infinite M1000 Pro microplate reader (Mänedorf, Zürich, Switzerland). This assay was performed in duplicate and repeated three times.

### 2.8. Inner Membrane Permeabilization Assay

Due to a deficiency in lactose permease, the cytoplasmic β-galactosidase of *Escherichia coli* ML35 (ATCC 43827) was released into the medium and it subsequently hydrolysed 2-nitro-phenyl β-D-galactopyranoside (ONPG, N1127, Sigma, Shanghai, China) to 2-nitrophenol (ONP) when the inner membrane was punctured. The bacteria were cultured in nutrient broth (NB, 23400, BD, Franklin Lakes, NJ, USA), grown to mid-logarithmic phase and diluted to 1 × 10^7^ CFU/mL in 10 mM sodium phosphate buffer containing 1% NB. Eighty microlitres of the bacteria was incubated with 10 µL of HD5 and RC18 and 2.5 mM ONPG at 37 °C for 2 h. The absorbance at 405 nm was reported at 5 min intervals, and the experiment was performed in duplicate and repeated three times.

### 2.9. Scanning Electron Microscopy (SEM)

MDRAB and MRSA were grown to mid-logarithmic phase in MHB and diluted to 1 × 10^8^ CFU/mL. A 1 mL aliquot of the bacteria was centrifuged and resuspended in 50 µL of sodium phosphate buffer (pH = 7.4). Five microlitres of HD5 and RC18 were then added at final concentrations of 60 and 100 µg/mL. The culture was then incubated at 37 °C with shaking at 130 rpm for 30 min. Ten microlitres of the mixture was pipetted to coat a coverslip, which was then dried at room temperature. The coverslips were processed with 2.5% glutaraldehyde, dehydrated with gradient concentrations of ethanol, and desiccated with tert-butyl alcohol. A Zeiss (Oberkochen, Germany) Crossbeam 340 scanning electron microscope was used to observe the bacterial morphology.

### 2.10. Molecular Dynamics Simulation (MDS)

All-atom MDSs were performed with the CHARMM36 force field implemented in Gromacs 2019.6 [24,25]. The structural model of RC18 was predicted by AlphaFold2 [26]. The system contained RC18, a bilayer of phospholipids, water (TIP3P model) and ions (150 mM KCl). A typical bilayer membrane was composed of 42 molecules of MAIPG, 54 molecules of AIPG, 22 molecules of AIPE, 14 molecules of DPPG, and trace amounts (<10 molecules each) of DPPE and PAICL2 [27,28,29]. MAIPG, PG (14:0/a15:0); AIPG, PG (a15:0/i15:0); AIPE, PE (a15:0/i15:0); DPPG, PG (16:0/16:0); DPPE, PE (16:0/16:0); PAICL2, CL (1’-[16:0/a15:0],3’-[16:0/a15:0]) (Figure S2).The total number of atoms was 68,875. RC18 and the membrane were solvated in a tetragonal box the size of 84.1 × 84.1 × 104.1 Å^3^. The Nose-Hoover thermostat (303.15 K) and Parrinello-Rahman isotropic NPT ensembles were adopted with h-bond LINCS constraints. Verlet cut-off schem and nblist update frequency of 20 was adopted in neighbor searching procedures with xyz periodic boundary conditions; options for electrostatics and VdW calculations were PME (coulombtype) and Cut-off (vdwtype, 1.2 rvdw). The input Gromacs file (.gro) was provided in the Supplementary Materials. First, energy minimizations were performed to relieve unfavourable contacts, followed by 10 ns of equilibration steps. Subsequently, simulations were performed using three independent trajectories for 100 ns. Representative conformations were created from each trajectory and analysed by *gmx hbond* tools.

### 2.11. Toxicological Evaluation

Human umbilical vein endothelial cells (HUVECs) obtained from the Chinese Academy of Sciences (CAS, Shanghai, China) were cultured in RPMI 1640 medium with 10% foetal bovine serum and seeded in a 96-well microtiter plate at a density of 5 × 10^4^ cells per well. RC18 was added after 24 h at concentrations of 12.5, 25, 50, 100, and 200 μg/mL. Cells were incubated at 37 °C for 24 h, and cell survival was determined using a cell counting kit-8 (CCK-8, Dojindo, Shanghai, China). The haemolysis of RC18 was evaluated as previously described [30]. Melittin (12.5 and 25 μg/mL) was utilized as the positive control. The absorbance of the blood supernatant obtained by centrifugation at 10,000× *g* for 5 min was measured at 570 nm. For in vivo toxicological evaluation, twelve 8-week-old female BALB/c mice (18–22 g) were randomly divided into 2 groups (n = 6). RC18 (12 mg/kg) was given every two hours by intraperitoneal injection. After 8 h, mouse blood was collected for alanine aminotransferase (ALT) and aspartate aminotransferase (AST) detection. Additionally, mouse liver, spleen, and kidneys were collected by surgery for haematoxylin-eosin (HE) staining. Mice were cared for and treated in accordance with the NIH guidelines for the care and use of laboratory animals (NIH Publication no. 85e23 Rev. 1985). All mouse experiments were conducted with approval from the Animal Experimental Ethics Committee of the Third Military Medical University.

### 2.12. Mouse Peritoneal Infection Model

Thirty female 8-week-old BALB/c mice were randomly divided into three groups (n = 10), and a total of 9 × 10^7^ CFU of MRSA was intraperitoneally administered. RC18 (12 mg/kg) was given by intraperitoneal injection 0.5, 2.5, and 4.5 h after bacterial infection. CTX (1.6 mg/kg) was injected 0.5 h post infection as the negative control. Mouse survival was monitored for 5 days. To determine bacterial colonization in vivo, another eighteen mice were randomly divided into three groups (n = 6). After antibiotic treatment, the mice were euthanized 8 h post infection. Two millilitres of saline was used to rinse the mouse peritoneal cavity, and the lavage fluid was collected for bacterial detection. Furthermore, the liver and spleen were collected by surgery and homogenized in 1 mL of saline. One hundred microlitres of the fluid was coated on a MHB agar plate containing 10 μg/mL CTX, and the bacterial loads were counted after a 24 h culture at 37 °C.

### 2.13. Statistical Analysis

Statistically significant differences (*P*) between groups were calculated with GraphPad Prism 8 (GraphPad Software Inc., La Jolla, CA, USA) using the unpaired two-tailed Student’s *t* test and one-way analysis of variance (ANOVA). *P* < 0.05 was defined as statistically significant.

## 3. Results and Discussion

### 3.1. AMP Design and Antimicrobial Assessment

To investigate the role of the γ-core structure in the antibacterial action of HD5, we extracted the amino acid sequences from Ser15 to Arg32 and synthesized the SL18 peptide (Figure 1A). A virtual colony count assay was then conducted, and the results revealed that SL18 was virtually inactive against *S. aureus* (ATCC 25923, Figure 1B) and *A. baumanii* (ATCC 19606, Figure 1C) at concentrations up to 8 μM, possibly due to the structure-dependent antibacterial action of HD5 [31,32]. Two intramolecular disulfide pairings were thus introduced to mimic the γ-core structure in stereo to generate another peptide termed SC18 [33]. Along with the structure optimization, less random coil structures were observed in SC18 than in SL18, as indicated by decreased signal intensity at 200 nm in CD spectroscopy (Figure 1D). As a result, the antibacterial property of SC18 was markedly improved relative to that of SL18, particularly against *S. aureus* (Figure 1B). After 1 h of incubation, the average antibacterial efficiency of 8 μM SC18 against *S. aureus* and *A. baumannii* was 95.8% and 62.3%, respectively. This activity could be because the defensin activity against Gram-positive bacteria was more dependent on peptide conformation than against Gram-negative bacteria [14,34].

Nevertheless, because of the simplified structural, SC18 was still less efficient than HD5 in bacterial killing. As Arg is highly selected for human α defensins from an evolutionary perspective [16], and because charge-reversal mutation through Arg introduction can compensate for the influence of structural deficiency [22], we replaced the only electronegative residue of SC18, Glu^7^, with Arg for functional improvement. The E7R mutation enhanced the antibacterial potency of SC18 against both *S. aureus* and *A. baumannii*. The resulting product even exhibited stronger efficacy against *A. baumannii* than HD5 at high concentrations (Figure 1C). We next substituted three electroneutral Ser residues (Ser^1^, Ser^3^, and Ser^8^) in E7R-SC18 with Arg to generate RC18 (Figure 1A) and this was encouraging. The RC18 peptide killed approximately 94.6% and 98% of *A. baumannii* and *S. aureus* at 1 μM, respectively, which were higher than the killing of HD5 (23% for *A. baumannii* and 89.8% for *S. aureus*). Results of the broth microdilution method revealed that RC18 was more efficient than HD5 in killing the two standard strains and another two resistant bacteria, MDRAB and MRSA (Table 1). Notably, the MIC of RC18 at killing MRSA was 40 μg/mL, which was eight- and two-fold lower than that of HD5 and T7E21R-HD5 [35], respectively, indicating that RC18 could be a potential therapeutic for MRSA infection.

### 3.2. RC18 Attraction to the Bacterial Membrane

**Figure 2:**
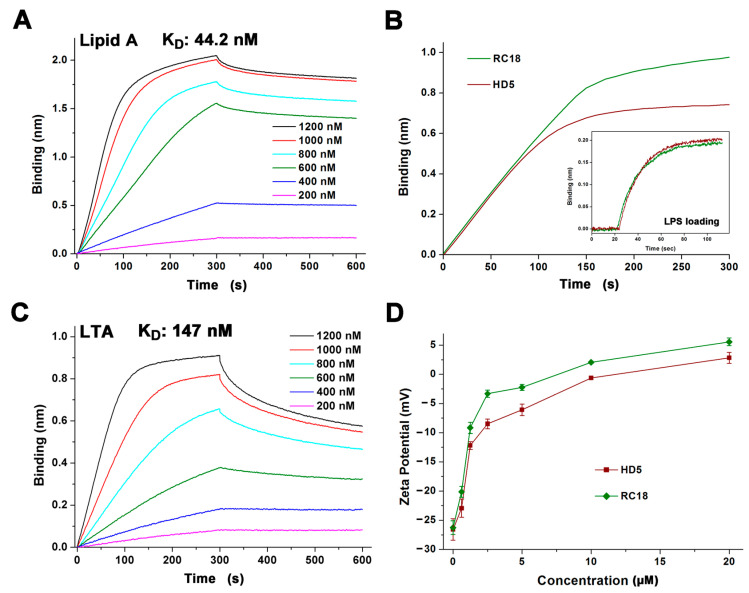
(**A**) Binding kinetics of RC18 to lipid A. Peptides were prepared in 10 mM sodium phosphate buffer (pH 7.4) at concentrations of 200, 400, 600, 800, 1000 and 1200 nM. (**B**) Thickness of the bond between 1000 nM HD5 and RC18 to immobilized LPS on AR2G biosensors. The results showed that more RC18 peptides were recruited to LPS than that of HD5 peptides. (**C**) Binding kinetics of RC18 to LTA. (**D**) Surface charge of microbes exposed to HD5 and RC18 at concentrations of 0.625, 1.25, 2.5, 5, 10, and 20 μM. The results are shown as the mean ± SD.

The electrostatic interaction between cationic AMPs and anionic membranes containing molecular targets such as lipid A and LTA on Gram-negative and Gram-positive bacteria, respectively, is known as the initial step of AMP-mediated bacterial killing [15,36]. To better understand the antibacterial action of RC18, we initially conducted BLI to analyse the association of the peptide with lipid A and LTA that was loaded on the AR2G biosensors. HD5 interacts with lipid A and LTA at an average binding affinity of 120 and 220 nm, respectively [22]. RC18 bound to lipid A in a dose-dependent manner in 10 mM sodium phosphate buffer with an equilibrium dissociation constant (K_D_) of 44.2 nM (Figure 2A), which was 2.7-fold lower than that of HD5 interacting with lipid A. As lipid A is the innermost element of LPS, a major component of the outer membrane of Gram-negative bacteria, a larger number of RC18 peptides were recruited to the immobilized LPS than that of HD5 at 1000 nM (Figure 2B). Similarly, RC18 bound to LTA at 147 nM (Figure 2C), an affinity 1.5-fold lower than that of HD5, which contributed to the higher surface charge of RC18-treated *S. aureus* when compared to microbes exposed to equivalent concentrations of HD5 (Figure 2D).

### 3.3. Bacterial Membrane Disruption Induced by RC18

**Figure 3:**
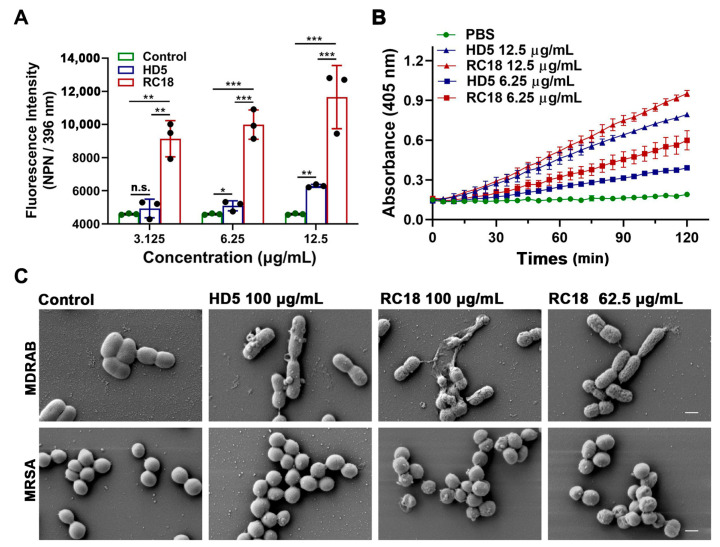
(**A**) Outer membrane infiltration assay. The fluorescence intensity of NPN after excitation at 396 nm is shown. The results are presented as the mean ± SD. *, *P* < 0.05; **, *P* < 0.01; ***, *P* < 0.001. n.s., not significant. (**B**) Inner membrane permeabilization assay. The yield of the yellow product *ο*-nitrophenol was detected at 405 nm. The results are presented as the mean ± SD. (**C**) SEM images demonstrated the morphological changes in MDRAB and MRSA exposed to HD5 and RC18. The scale indicates 500 nm.

AMPs successively infiltrate bacterial outer and inner membranes following attraction to the surface [31]. To investigate the influence of RC18 on the bacterial membrane, we initially utilized 1-N-phenylnaphthylamine (NPN), a hydrophobic dye that can incorporate into damaged membranes and fluoresce upon excitation at 350 nm, to indicate the infiltration into outer membranes [37]. After wavelength scanning from 380 to 450 nm, we determined that NPN exhibited a maximum emission at approximately 396 nm (Figure S3). As shown in Figure 3A, the fluorescence intensity of MDRAB exposed to 6.25 and 12.5 μg/mL HD5 at 396 nm was significantly higher than that of the control group, which was consistent with our previous findings [15]; meanwhile, RC18 induced markedly increased fluorescence intensity than HD5 at equivalent concentrations, indicating that RC18 was superior to HD5 in outer membrane infiltration. Moreover, a 2-nitro-phenyl β-D-galactopyranoside (ONPG) hydrolytic assay was performed to evaluate inner membrane permeabilization elicited by RC18 treatment. In line with the results from the NPN uptake assay, RC18 disrupted the inner membrane in a dose-dependent manner (Figure 3B). Based on the yield of ο-nitrophenol, RC18 induced disintegration of the inner membrane to a greater extent than did the equivalent concentration of HD5. SEM results verified that both MDRAB and MRSA lost their membrane integrity after 30 min of incubation with the peptide, and 62.5 μg/mL RC18 (28 μM) disrupted the membrane to a greater extent than 100 μg/mL HD5 (28 μM), based on the unsmooth membrane, cytoplasmic leakage, and microbial aggregation seen after RC18 treatment (Figure 3C).

### 3.4. MDS Modelling of the Membrane Disruption by RC18

**Figure 4:**
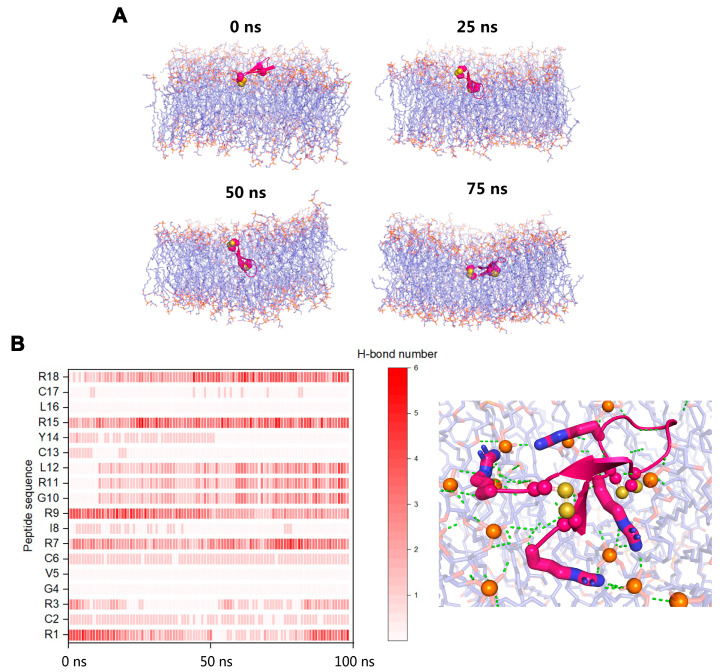
(**A**) Snapshots of RC18 penetrating the bacterial membrane and causing deformations. (**B**) Arg residues mediated most of the polar interactions between RC18 and the phospholipid head groups. Arg1, Arg7, Arg15, and Arg18 are highlighted as sticks. The disulfide bonds and the nearby phosphorus atoms (orange colour) are represented as spheres. Polar interactions are highlighted as green dashes.

To elucidate the molecular mechanisms by which RC18 disrupts bacterial membranes, we performed all-atom MDSs. At the initial (0 ns) equilibrated conformation, RC18 attached to the bilayer surface. When RC18 infiltrated the phospholipids, the overall shape of the membrane deformed, as demonstrated by the 50 ns and 75 ns snapshots (Figure 4A). Different from the conformational changes of flexible peptides in various media [31,38], RC18 kept the β hairpin structure throughout the simulations, owing to the stabilization of the intramolecular disulfide bonds. Among the simulated trajectories, residues Arg1, Arg7, Arg9, Arg15, and Arg18 mediated the majority of the polar interactions between RC18 and the phospholipid head groups (Figure 4B), possibly due to the multiple hydrogen bonds donated by the guanidinium group of Arg [39]. Additionally, hydrophobic residues Val5 and Leu16 were associated with apolar groups on the membrane, and this was the mechanism by which RC18 deeply infiltrated the membrane.

### 3.5. Toxicological Evaluation of RC18

**Figure 5:**
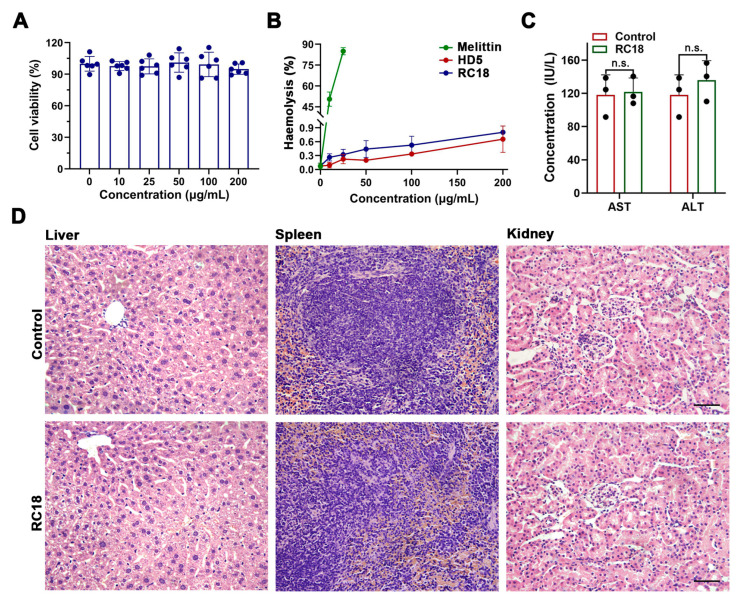
(**A**) Survival of HUVECs exposed to RC18 at various concentrations. Dots indicate individual results from three independent experiments. (**B**) Haemolysis of the peptides with melittin applied as a positive control. The results are presented as the mean ± SD. (**C**) Levels of AST and ALT in the plasma of mice in the presence or absence of RC18 treatment. Dots indicate individual results from three mice. n.s., not significant. (**D**) HE staining of the liver, spleen, and kidney of RC18-treated mice. Mice in the control group were treated with PBS. The scale indicates 200 μm.

Considering that the hydrophobic residues contribute to the membrane interactions of RC18, and because hydrophobic residues may account for the virulence of certain AMPs [40,41], we next asked if RC18 was toxic to host cells. Actually, the important cytotoxicity dose often hampers the development of many potent AMPs, such as melittin and latarcin [42,43], into antibiotic candidates. To assess the cytotoxicity of RC18, we incubated HUVECs with the peptide at increasing concentrations. The results showed that RC18 had less of an effect on cell survival after 24 h at doses up to 200 μg/mL (Figure 5A), a value approximately 10-fold higher than the MIC of RC18 in MRSA. Furthermore, RC18 was far less detrimental to erythrocytes than melittin (Figure 5B). The percent haemolysis of 200 μg/mL RC18 was under 1%, whereas 25 μg/mL melittin caused haemolysis of more than 80%. For in vivo toxicity evaluation, BALB/c mice were given 12 mg/kg RC18 by intraperitoneal administration. Mouse blood was collected 12 h after injection for the detection of ALT and AST content. As displayed in Figure 5C, RC18 treatment did not induce early liver injury. Additionally, RC18 did not induce the changes of blood urea nitrogen and serum creatinine (Figure S4), suggesting its biosafety to kidney. Furthermore, the liver, spleen and kidneys were collected after seven days. HE staining revealed no pathological changes in the tissues (Figure 5D). These data collectively indicated the biocompatibility of RC18, providing a good foundation for further in vivo antibacterial evaluation.

### 3.6. In Vivo Antibacterial Evaluation of RC18

**Figure 6:**
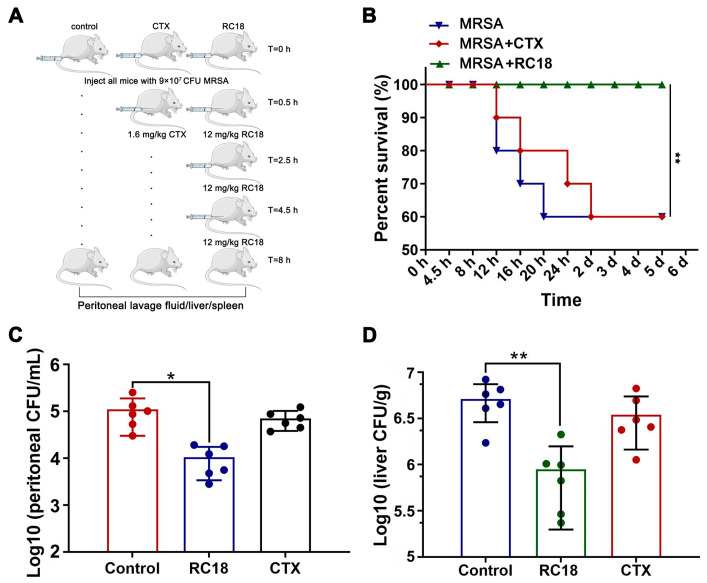
(**A**) Image depicting the mouse peritoneal infection experiment. (**B**) Survival curve of the infected mice after treatment with RC18 and CTX. **, *P* < 0.01. (**C**) Bacterial loads in mouse peritoneal lavage fluid. The results are presented as the mean ± SD. *, *P* < 0.05. (**D**) Bacterial colonization in mouse liver. The results are presented as the mean ± SD. **, *P* < 0.01.

To assess the efficacy of RC18 in vivo, a mouse peritoneal infection model was established. BALB/c mice were given a total of 9 × 10^7^ CFU of MRSA via intraperitoneal injection, and RC18 was administered at 12 mg/kg using a dosage regimen as recently described [44]. A single injection of 1.6 mg/kg CTX 0.5 h after bacterial invasion was utilized as the control treatment (Figure 6A). Mouse survival was monitored for 5 days. The results showed that 40% of the mice given PBS or CTX died after 5 days, whereas the mice treated with RC18 remained alive (Figure 6B, *P* < 0.01). Further investigation revealed that in the peritoneal lavage fluid of the untreated mice, we detected approximately 1 × 10^5^ CFU of resistant bacteria (Figure 6C), which was significantly higher than that in the mice treated with RC18 (*P* < 0.05). Additionally, RC18 markedly lowered the bacterial loads in the liver (Figure 6D, *P* < 0.01) and spleen (Figure S5). Comparatively, CTX injection did not influence bacterial colonization, either in the liver or in the spleen. It is plausible that RC18 is superior to CTX in suppressing MRSA infection in vivo, and therefore RC18 is an inspiring antibiotic candidate.

Collectively, we designed and evaluated a potent peptide antibiotic based on the γ-core motif of HD5. By binding to and penetrating bacterial membranes, this biocompatible peptide termed RC18 efficiently killed bacteria in vitro and in vivo, thus providing a promising antibiotic candidate to treat infection caused by resistant bacteria. However, because of the focus on membrane interactions, the study has shortcomings. It is known that AMPs may inactivate microbes through an intracellular mechanism in addition to membrane disruption. For instance, proline-rich AMPs can kill bacteria by binding to the ribosome and disrupting protein synthesis [45,46], and polyphemusin-I, a β-hairpin AMP from horseshoe crabs, may target bacterial nucleic acid-associated proteins [47]. We previously also found that HD5d5, a flexible derivative of HD5, could translocate into the cytoplasm of MDRAB and attenuate the activities of superoxide dismutase and catalase, causing a lethal accumulation of reactive oxygen species [15]. As a portion of the MDRAB and MRSA cells were not fully collapsed after exposure to RC18 (Figure 3C), we speculated the existence of intracellular targets for RC18 to kill bacteria. Therefore, additional research is needed to probe the events of RC18 following membrane penetration in bacterial killing.

## 4. Conclusions

RC18, a potent peptide antibiotic that acts against resistant bacteria, was designed by extracting the γ-core structure of HD5 and was further characterized in this study. Owing to the conformational simplification and Arg introduction, RC18 is superior to HD5 in facile preparation and bacterial killing. This peptide is attracted to the bacterial membrane through electrostatic interactions with anionic lipid A and LTA, allowing subsequent membrane destruction. The Arg residues are not only crucial for the recruitment of RC18 to bacterial membranes, but they are also instrumental to membrane penetration. RC18 is biocompatible and effective at eliminating MRSA in vivo. Considering that the development of effective antibiotics for resistant bacterial infections is a pressing clinical need, we believe that RC18 may be a promising therapeutic candidate.

## Figures and Tables

**Table 1:** MICs (μg/mL) of antimicrobial agents required to eliminate *A. baumannii* and *S. aureus*, as calculated using the broth microdilution method.

Antimicrobial Agents	*A. baumannii*		*S. aureus*	
	MDRAB	ATCC19606	MRSA(ATCC43300)	ATCC25923
HD5	320 (89.3 μM )	320 (89.3 μM )	160 (44.7 μM )	160 (44.7 μM )
RC18	160 (71.6 μM)	160 (71.6 μM)	40 (17.9 μM)	40 (17.9 μM)
CTX ^a^	640	10	>640	≤2.5
CIP ^b^	320	≤2.5	160	≤2.5

^a^, CTX, cefotaxime; ^b^, CIP, ciprofloxacin.

## Data Availability

All data generated and analyzed during this study are included in this published article and its Supplementary Materials file.

## Supplementary Materials

The following supporting information can be downloaded at: https://www.mdpi.com/article/10.3390/membranes13010051/s1, Figure S1: Purities and molecular weights of the peptides; Figure S2: Structures of MAIPG, AIPG, AIPE, and PACL2; Figure S3: NPN uptake assay determining the membrane destruction of RC18; Figure S4: Blood urea nitrogen and serum creatinine contents in RC18–treated mice; Figure S5: Bacterial colonization in mouse spleen; and the input Gromacs file (.gro) for MDS.
