# Supplementary material for: γ-Core Guided Antibiotic Design Based on Human Enteric Defensin 5
Source: Membranes (Basel). 2022 Dec 31;13(1):51. doi: 10.3390/membranes13010051 (PMC9862697; doi:10.3390/membranes13010051)
Supplement: Supplementary file 1 [file membranes-13-00051-s001.zip › membranes-2083655-supplementary.pdf]

membranes

Supplementary Materials

γ -core Guided Peptide Antibiotic Design based on Human Enteric Defensin 5

Gaomei Zhao [1,†], Changsheng Jia [2,†], Cheng Zhu [3,†], Minchao Fang [3], Chenwenya Li [1], Yin Chen [1], Yingjuan He [1], Songling Han [1], Yongwu He [1], Jining Gao [1], Tao Wang [1], Cheng Wang [1,*] and Junping Wang [1,*]

[1] State Key Laboratory of Trauma, Burns and Combined Injury, Institute of Combined Injury of PLA, Chongqing Engineering Research Center for Nanomedicine, College of Preventive Medicine, Third Military Medical University, Chongqing 400038, China
[2] Department of Pharmacy, Southwest Hospital, Third Military Medical University, Chongqing 400038, China
[3] Tianjin Key Laboratory of Function and Application of Biological Macromolecular Structures, School of Life Sciences, Tianjin University, Tianjin 300072, China
* Correspondence: wangctmmu@126.com (C.W.); wangjunping@tmmu.edu.cn (J.W.)
† These authors contributed equally to this work.

Contents:

Figure S1. Purities and molecular weights of the peptides
Figure S2. Structures of MAIPG, AIPG, AIPE, and PACL2
Figure S3. NPN uptake assay determining the membrane destruction of RC18
Figure S4. Blood urea nitrogen and serum creatinine contents in RC18-treated mice
Figure S5. Bacterial colonization in mouse spleen

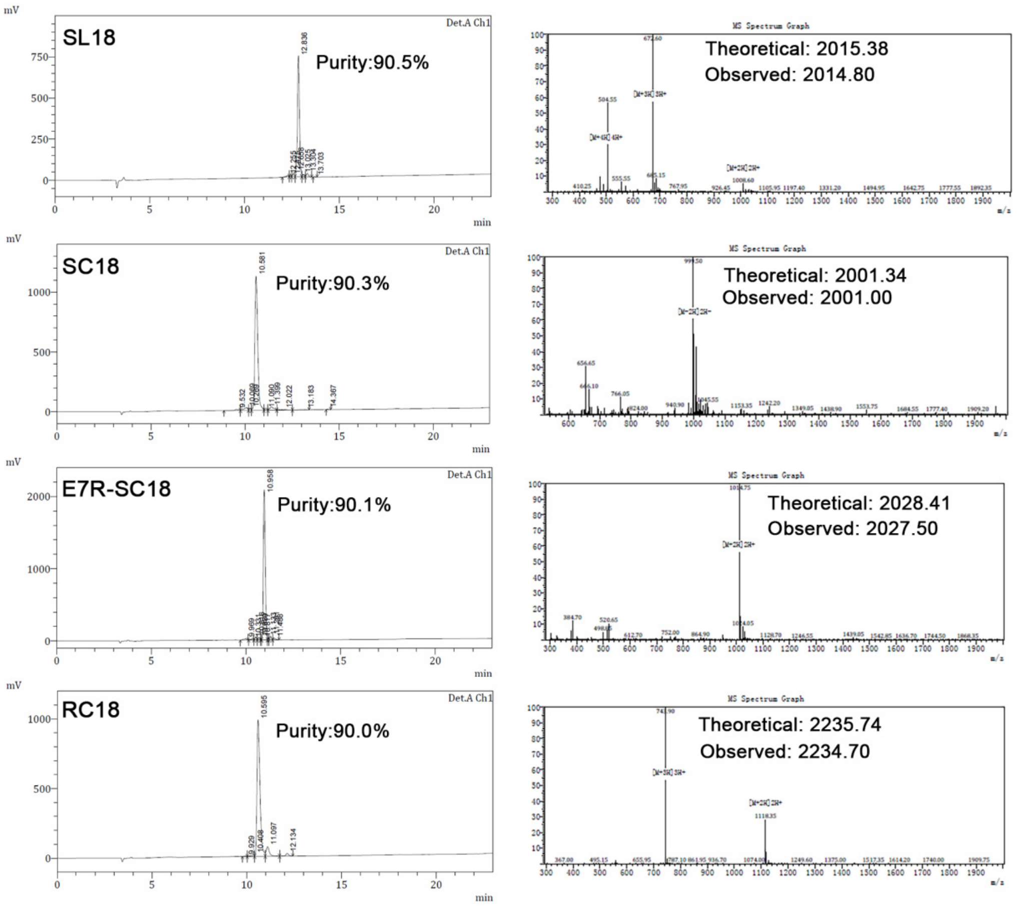

Figure S1. Purities and molecular weights of the peptides. The purities of peptides were over 90%. The determined molecular weights of peptides matched closely with their theoretical values.

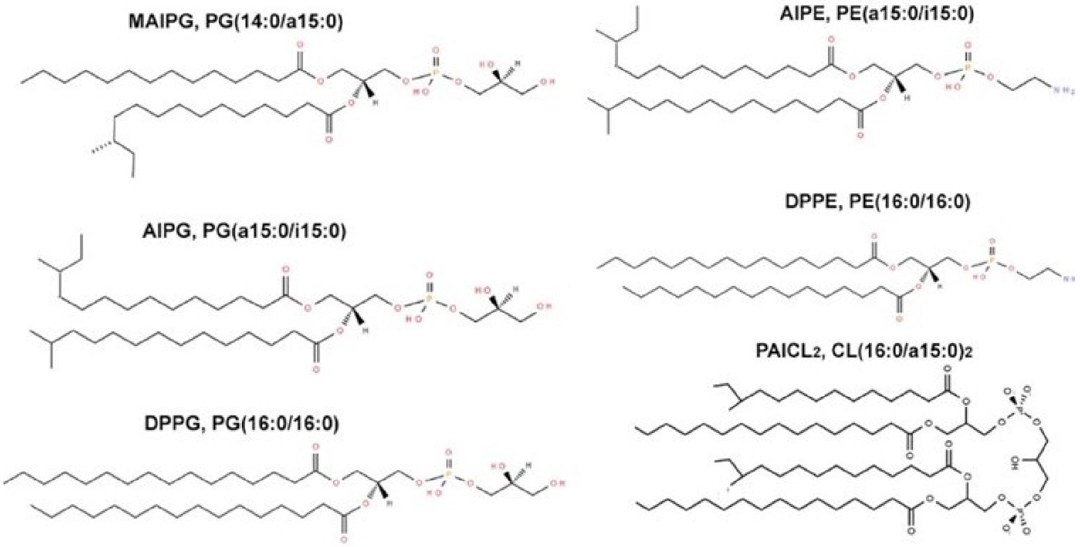

Figure S2. Structures of MAIPG, AIPG, AIPE, and PACL2.

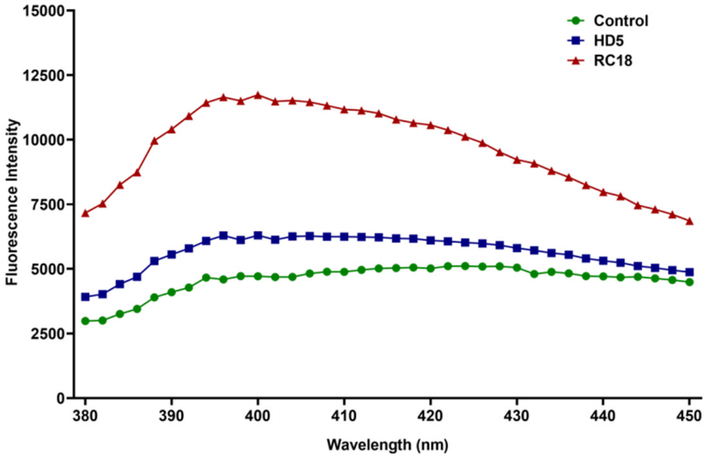

Figure S3. NPN uptake assay determining the membrane destruction of RC18. The NPN dye was excited at 350 nm, and the increased fluorescence intensity was measured from 380 to 450 nm with an interval of 2 nm. The results are presented as the mean ± SD.

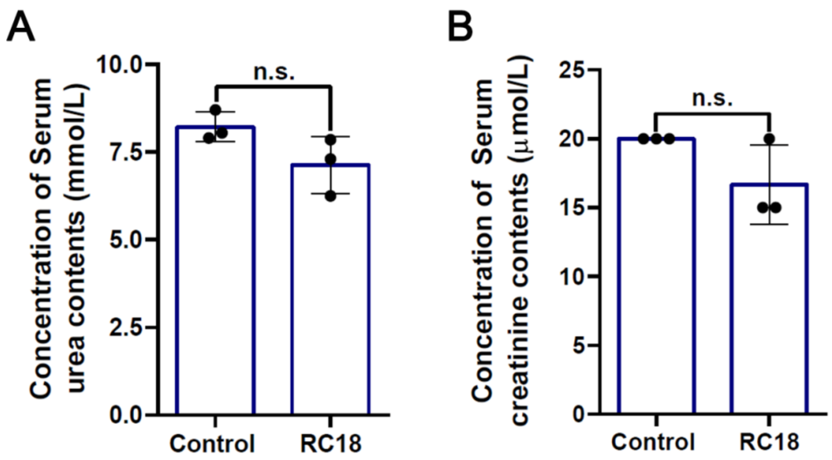

Figure S4. Blood urea nitrogen and serum creatinine contents in RC18-treated mice. (**A**) Levels of Blood urea nitrogen. (**B**) Levels of serum creatinine contents in mice untreated or treated with RC18.The results are presented as the mean ± SD. n.s., not significant.

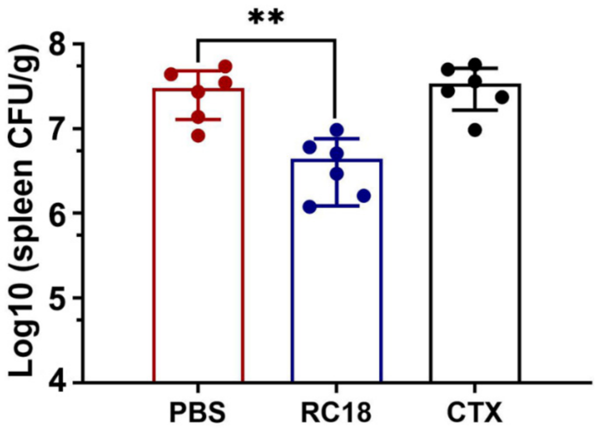

Figure S5. Bacterial colonization in mouse spleen. The results are presented as the mean ± SD. **, *P* < 0.01.
